# Nutritional Issues among Children with Duchenne Muscular Dystrophy—Incidence of Deficiency and Excess Body Mass

**DOI:** 10.3390/nu16132143

**Published:** 2024-07-04

**Authors:** Edyta Wernio, Eliza Wasilewska, Sylwia Czaja-Stolc, Karolina Śledzińska, Jolanta Wierzba, Agnieszka Szlagatys-Sidorkiewicz, Sylwia Małgorzewicz

**Affiliations:** 1 Department of Clinical Nutrition and Dietetics, Medical University of Gdansk, 80-210 Gdansk, Poland; edyta.wernio@gumed.edu.pl (E.W.); sylwia.malgorzewicz@gumed.edu.pl (S.M.); 2 Department of Pulmonology & Allergology, Medical University of Gdansk, 80-210 Gdansk, Poland; eliza.wasilewska@gumed.edu.pl; 3 Department of Paediatrics, Haemathology and Oncology, Medical University of Gdansk, 80-210 Gdansk, Poland; karolina.sledzinska@gumed.edu.pl; 4 Department of Internal and Paediatric Nursing, Medical University of Gdansk, 80-210 Gdansk, Poland; jolanta.wierzba@gumed.edu.pl; 5 Department of Paediatrics, Gastroenterology, Allergology & Paediatric Nutrition, Medical University of Gdansk, 80-210 Gdansk, Poland; agnieszka.szlagatys-sidorkiewicz@gumed.edu.pl

**Keywords:** nutritional status, Duchenne muscular dystrophy, screening nutritional assessment, malnutrition, overweight

## Abstract

The progression of Duchenne muscular dystrophy (DMD)requires the assessment of nutritional disturbances at each stage of the disease. The purpose of this study was to assess the nutritional status in various ages of boys with DMD using screening and in-depth evaluation methods. Body composition by Dual X-ray Absorptiometry (DXA), basal metabolic rate (BMR) by indirect calorimetry, a questionnaire of nutritional status—Pediatric Nutrition Screening Tool (PNST)—and laboratory parameters were performed. In the cohort of 93 boys aged 8.54 (5.9–12.6 years), inappropriate nutritional status occurred in 41.8% of boys (underweight 11.8%, overweight 16.0%, and obesity 14.0%). In the 10–13 age group, the occurrence of overweight and underweight was the highest. Based on PNST, 15.1% of patients were at nutritional risk (≥2 points)—the most in the 14–17 age group (29%). A negative correlation was identified between PNST and z-scores of body weight, BMI, and FFMI (r Spearman = −0.49, −0.46, and −0.48, respectively; *p* < 0.05). There were no differences between BMR results from indirect calorimetry and calculations from the Schofield formula for any age group. In obese boys, the caloric requirement in indirect calorimetry was significantly lower than that indicated by the calculations according to the Schofield formula (*p* < 0.028). Inappropriate nutritional status occurred in almost half of the children with DMD. The age group in which nutritional disorders were most frequently identified was 10–13 years old. PNST could be considered a tool for screening malnutrition after testing a larger group of DMD patients.

## 1. Introduction

**Figure 1:**
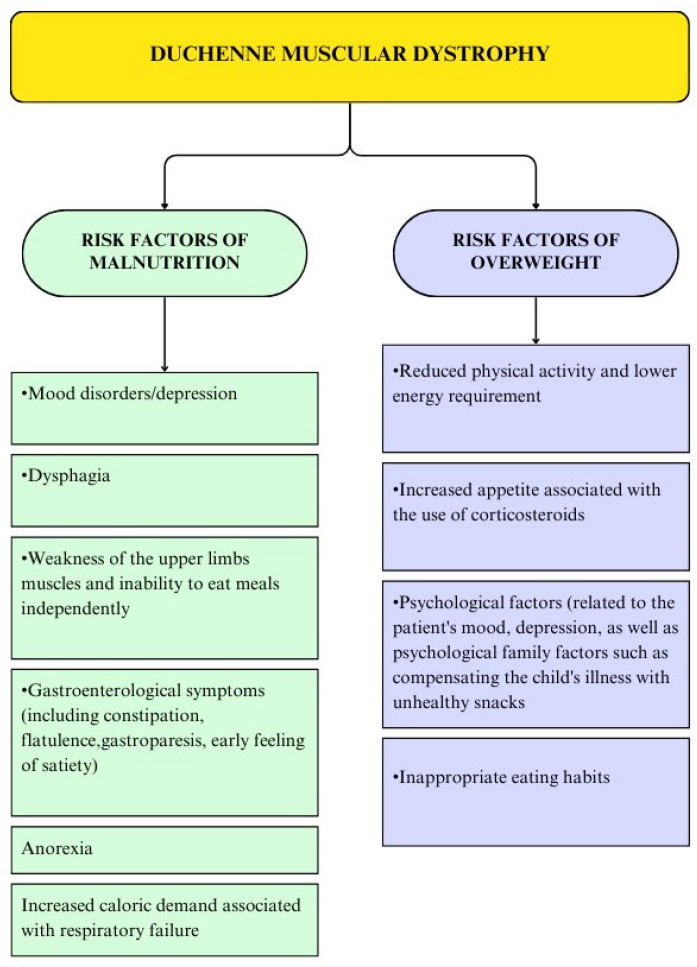
Risk factors of nutritional status disorders in children with DMD.

Duchenne muscular dystrophy (DMD) is a muscle-wasting disease with a severe progressive course. This X-linked recessive genetic disorder affects less than 7 cases per 100,000 males [1]. Pathological changes in the organism are irreversible and affect skeletal as well as respiratory, cardiac, and gastrointestinal tract function. The first symptoms of degenerative transitions are diagnosed between the ages of 2 and 6. Teenage children lose mobility, and, in adulthood, they require mechanical ventilation, often needing enteral nutrition [2,3]. Multidisciplinary medical care for a patient with DMD has contributed to the extension of the average life expectancy. Broomfield et al. proved that patients born after 1990 have a median life expectancy of 28.1 years, while the median survival age from pre-1970 births was 18.3 years [4]. An important part of overall DMD patient care is the assessment and management of nutritional status. Both excess body mass and undernutrition can be diagnosed among children with DMD, which is related to changes in body composition, impaired respiratory and cardiac functions, as well as steroid therapy and its potential effect on weight gain (Figure 1) [5,6].

The assessment of nutritional status is conducted using anthropometric measurements, body composition, and caloric requirements. Patients at an advanced stage of the disease or living at a considerable distance from the center may have a delayed diagnosis of abnormal nutritional status and thus delayed treatment. In clinical practice, there are simple, quick screening methods that can be used by caregivers of children, primary care physicians, and other specialists, such as relating weight, height, and BMI to percentile charts, asking about unintended weight loss, and/or loss of appetite. In case of an incorrect screening result, consultations with specialists (dietitian, physicians, psychologist, etc.) should be obligatory to further assess the nutritional status using more advanced methods, including, among others, body composition analysis, biochemical tests, examining eating behavior, as well as identifying the causes of nutritional abnormalities (e.g., gastrointestinal disturbances, swallowing disorders, depression, increased caloric demand related to respiratory failure, and lack of independence in eating).

The paper will present selected anthropometric parameters, a questionnaire assessment, as well as body composition and laboratory tests of children with DMD from various age groups. The aim of the study was also the identification of the age group where nutritional disturbances are most common. 

## 2. Materials and Methods

### 2.1. Study Design

This observational, prospective study, conducted from January 2019 to December 2022, was created to investigate the nutritional status of the cohort of DMD patients. Participants were recruited from the Rare Diseases Center of the University Clinical Center in Gdansk, Poland. This center belongs to the TREAT- NMD Alliance Neuromuscular Network and is accredited by the World Duchenne Organization. The Independent Bioethical Committee of the Medical University of Gdansk’s approval for the study was obtained (NKBBN/105/2018).

### 2.2. Participants

The following inclusion criteria were taken into consideration: (1) confirmation of DMD diagnosis according to current guidance [2]; (2) age ≥4 years and <18 years; (3) a willingness to cooperate; and (4) signed informed consent for participation in the study. Written informed consent was obtained from the parents or legal guardians of each patient. For patients over 16 years of age, consent was also obtained from the patients themselves, in addition to their parents.

Participants were divided into 4 groups according to the age criterion following the Polish Nutrition Standards guideline [7] with a slight modification to reflect the dynamics of the disease better: 4–6, 7–9, 10–13, and 14–17 years. 

### 2.3. Nutritional Status

The anthropometric measurements such as weight, height, body mass index (BMI), body composition analysis by Dual X-ray Absorptiometry (DXA), basal metabolic rate (BMR) based on caloric requirements measured with indirect calorimetry, screening questionnaire assessment of the nutritional status—Pediatric Nutrition Screening Tool (PNST) [8]—and laboratory parameters were assessed in each patient. 

The PNST was designed to screen for the nutritional status of pediatric patients. This simple tool consists of four questions: (1) regarding unintentional weight loss; (2) poor weight gain in the last few months; (3) reduced food intake in the last few weeks; and (4) considering whether the child is underweight. Positive responses are scored; obtaining ≥2 points indicates the risk of nutritional disturbances and the need to expand the assessment of nutritional status [8]. The questionnaire assessment was performed by an experienced clinical dietitian and the first three answers were provided by the parents.

#### 2.3.1. Anthropometry and Body Composition Analysis

Body mass (kg) was measured using a mechanical column scale (Seca 711, Hamburg, Germany) for ambulatory and a chair scale (Seca 956, Hamburg, Germany) for non-ambulatory participants. 

A telescopic stadiometer (Seca 220, Hamburg, Germany) was used to assess height (cm). In non-ambulatory boys and those with pustular defects, segmental measurement of the body length was performed. In teenagers with severe contractures of the lower limbs, ulna-length methods were used to measure the distance between the point of the elbow and the midpoint of the prominent bone of the wrist on the left side [9]. Body length was measured three times, and the arithmetic average was calculated. 

BMI was calculated as body weight (kilograms) divided by the square of body height (meters). For the recognition of nutritional status, the National Growth Reference Data—OLAF study results for BMI were used [10]. Overweight was defined as a BMI ≥ 85 and <95 CC, obesity ≥ 95 CC, and underweight < 5 CC [11]. 

The DXA method, with a total body scanning time of 5 to 7 min, was performed by a specially trained radiologic technologist. The examination was conducted with the standard procedure and data on fat mass (FM) (%, kg, percentile); fat mass index (FMI)—body fat to height ratio (body fat mass in kilograms divided by height in meters squared); visceral fat mass (g); fat-free mass (FFM) (kg); fat-free mass index (FFMI)—fat-free mass in kilograms divided by height in meters squared; and bone mineral content (BMC) were obtained. Additionally, z-scores for FM, FFM, and FFMI were calculated.

#### 2.3.2. Caloric Requirements 

The caloric requirement was measured with indirect calorimetry and calculated using the Schofield formula [12]. The device Fitmate WM 2008100401 (Cosmed, Rome, Italy) was used to assess BMR. Before the analysis, participants were fasting and maintained a calm seated position for 15 min. During the analysis, after putting the mask on and securely covering their nose and mouth, they spent 15 min in a supine position. Calibration was conducted automatically for every measurement. 

#### 2.3.3. Biochemistry 

A blood sample was collected during hospitalization as a routine procedure. Data on the concentration of albumin (g/L), total protein (TP, g/L), alanine aminotransferase (ALT, U/I), aspartate aminotransferase (AST, U/I), gamma-glutamyl transpeptidase (GGT, IU/L), total bilirubin (mg/dL), total cholesterol (mg/dL), low-density lipoprotein cholesterol (LDL, mg/dL), high-density lipoprotein cholesterol (HDL, mg/dL), creatine phosphokinase (CPK, IU/L), lactate dehydrogenase (LDH, U/I), creatinine (mg/dL), iron (Fe, ug/dL), ferritin (ng/dL), hemoglobin (g/dL), glucose (mg/dL), vitamin D_3_ (25OH)D_3_ (ng/dL), calcium (mg/dL), and magnesium (mg/dL) were assessed. 

### 2.4. Statistical Analysis 

For statistical analysis, Statistica PL 13.3 for Windows and GraphPad Prism 8.4.3 were used. The Shapiro–Wilk test was employed to determine the distribution of variables. Data were presented as the mean ± standard deviation (SD) or median and interquartile range. Results were considered statistically significant with a *p*-value < 0.05. Depending on the data distribution, a Pearson and/or Spearman test analysis of correlations was conducted. The T-test or the U Mann–Whitney test were applied in a comparative analysis of the two groups. The ANOVA Kruskal–Wallis was used to compare the four age groups of patients along with Bonferroni’s post hoc test. Χ^2^ Pearson’s test was conducted to evaluate the association between categorized variables. The criterion for statistical significance was *p* < 0.05.

## 3. Results

A total of 93 boys with DMD (mean age 8.54 years; range 4.7–16.9) were evaluated; 66 were ambulatory, and 51 received steroid therapy. The boys were not treated with testosterone. The details of clinical information are presented in Table 1.

### 3.1. Anthropometry

Weight, height, and BMI increased in successive age groups (*p* < 0.001); however, there were no differences between groups when these parameters were calculated as centiles or z-scores (*p* > 0.05). There was a disparity between the mean values of weight (40 percentiles) versus height (9 percentiles) for the entire cohort and also for every age group (Table 2). 

**Figure 2:**
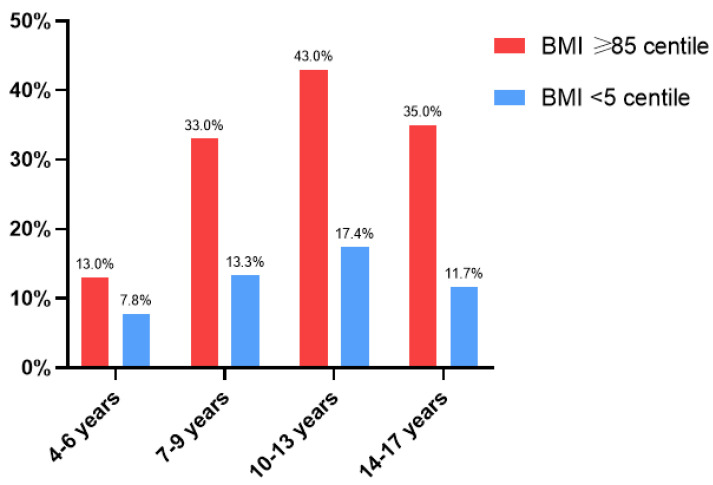
The percentage of patients with low and high BMI centiles in age groups.

Based on BMI centile, both undernourished and overweight patients occurred in all groups (but in different proportions). Undernutrition was diagnosed in 11.8% (n = 11) of children, overweight in 16.0% (n = 15), and obesity in 14.0% (n = 13). The group aged 10–13 years presented the highest number of overweight (43%) and underweight (17.3%) children compared to other groups. The percentage of patients with low and high BMI centiles in each of the age groups is presented in Figure 2.

### 3.2. Body Composition

FFM, FM, and visceral fat mass increased in successive age groups (*p* < 0.002). However, there were no differences between groups when these results were calculated as centiles or z-scores (*p* > 0.05) except for FM [z-score], which increased reaching the highest value in the 10–13 age group and then decreased (*p* < 0.009) (Table 2). 

There was no difference in FFMI [kg/m^2^] between groups (*p* > 0.05), but the z-score of FFMI increased in successive age groups (*p* < 0.029). Moreover, a negative relationship was observed between the z-score of FFMI and age (r = −0.64, *p* < 0.001), and it was positive with a percentile of BMI (r = 0.47, *p* = 0.002) as well as with doses (mg) of Deflazacort (r = 0.54, *p* = 0.023).

### 3.3. Caloric Requirements

The BMR increased with age, with the highest value for the 14–17 age group (Table 2). There were no significant differences between BMR results from indirect calorimetry and calculations from the Schofield formula for any age group. The correlation between both methods (indirect calorimetry and Schofield formulae) was high (r Spearman = 0.56, *p* < 0.05).

Importantly, in obese boys, the caloric requirement in indirect calorimetry was significantly lower than indicated by the calculations according to the Schofield formula (*p* < 0.028) (Table 3).

### 3.4. Pediatric Nutrition Screening Tool (PNST)

**Figure 3:**
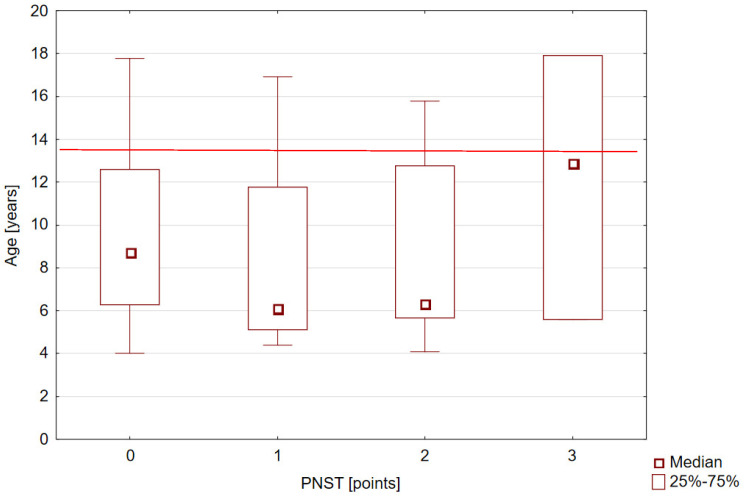
The Pediatric Nutrition Screening Tool median value in the study group.

Based on the PNST, 14 (15.1%) boys were at nutritional risk (PNST ≥ 2 points). In the 14–17 age group, as many as 29% of patients presented a risk of malnutrition (Table 2), but the highest median score was observed in the 10–13 age group (Figure 3).

Significant differences between the groups with PNST <2 and PNST ≥2 were observed. Boys being at nutritional risk according to the PNST presented lower body weight (*p* < 0.002), BMI (*p* < 0.001), FFMI [z-score/percentiles] (*p* < 0.04/*p* < 0.009), and FM [z-score] (*p* < 0.03) (Table 4). Moreover, a significant negative correlation between the PNST and z-scores of body weight, BMI, and FFMI was observed (r Spearman = −0.49; −0.46; and −0.48, respectively; *p* < 0.05)—Table 5.

### 3.5. Biochemical Results

The concentration of albumin and TP remained within a normal range in all groups, but two boys presented lower albumin < 38 g/L and four boys had CRP > 5 mg/dL. The mean level of the 25(OH)D_3_ was 23.6 ± 10.5 ng/mL; a deficiency or suboptimal concentration of vitamin D (<30 ng/mL) was present in 66 patients despite the supplementation. All laboratory parameters are presented in Table 6.

## 4. Discussion

The study aimed to assess the nutritional status in a cohort of DMD boys aged 4–17 years and identify the age group in which nutritional status disorders are most common. 

The most noteworthy result of the study was that almost half of the patients (41.8%) in our cohort presented abnormal nutritional status. Excess body weight was the most common abnormality (30% of boys), but also underweight affected 1 in 10 children (11.8%). Surprisingly, the boys with undernutrition or overweight were present in every age group but with different proportions. The most diverse group, with the largest number of overweight or underweight children, was the 10–13 age group. However, the highest number of patients (nearly one-third) with the risk of malnutrition (calculated according to PNST) was in the 14–17 age group.

### 4.1. Nutritional Status

DMD patients are a special group in which each person is at risk of obesity or malnutrition depending on the duration of the disease. As expected, our patients initially developed obesity, mainly of the central type. The reason is mainly steroid therapy which is an obesogenic factor and affects metabolic changes. Ectopic fat tissue develops lipotoxicity, causes mitochondrial dysfunction, and impairs the functioning of internal organs [13]. 

The first signs of malnutrition (decreased appetite/low food intake, decreased FFMI) were observed at about 13 years old, independently of BMI. A similar trajectory of changes in body weight over time can be seen in the reports of other researchers [14,15]. Billish N et al. proved that obesity develops until the age of 11 (50.6% were obese), after which the prevalence of excess body mass declines. After the age of 18, 20% of patients were malnourished [14]. According to Martigne L. et al., at the age of 13 years, 73% of patients were obese and 4% were underweight. Between the ages of 15 and 26, these proportions changed: 47% were obese and 34% developed malnutrition [15]. The authors of the cited papers suggest that being overweight in the DMD teenage years may be desirable, given the increased risk of malnutrition associated with disease progression. Therefore, it should be investigated whether the BMI norms for the population of DMD patients should be more liberal [14,15].

### 4.2. Screening Assessment of Nutritional Status

Early diagnosis of malnutrition is a crucial element of care. Based on BMI centiles, undernutrition was diagnosed in 11.8% of children, and considering PNST, 15.1% of patients were at nutritional risk. It is worth emphasizing that PNST results were negatively correlated with muscle and fat mass z-score, as well as BMI z-score and BMI percentiles. It is advisable to consider this questionnaire as a screening tool for assessing the risk of malnutrition, and due to the simplicity of implementation, it may become a patient’s self-monitoring tool. It is worth examining the usefulness of this tool by other research teams. PNST has been studied in various age groups and various clinical situations, for example, in the work of Carter et al., where the study group consisted of children from 1 month to 17 years of age [16]. A questionnaire that is easy for patients to complete is an important element of self-assessment. The PNST seems to be comfortable to self-administer. What is interesting is that, in our study, PNST identified the risk of malnutrition in each age group regardless of BMI centile. Therefore, a high BMI centile did not exclude the risk of malnutrition. 

PMST consists of four questions and takes into account unintentional weight loss; poor weight gain in the last few months; reduced food intake in the last few weeks; and considering whether the child is underweight [8]. Therefore, after dietitians conduct training for parents and patients, the tool could be an important element of the self-monitoring of nutritional status. In children with DMD, anthropometric assessment and body composition measurements are recommended [2]. In our opinion, screening methods are employed insufficiently since they could help assess nutritional risk, as in other patient groups; moreover, they could be performed at home or during an outpatient visit. Body composition measurements are not possible in all children due to availability and feasibility. It seems crucial to recognize malnutrition despite a high BMI, which, as our study shows, appears in every age with a peak around the age of 10–13. As expected, the preponderance of children with poorer nutrition status was noted in the oldest age group of patients (29% showed ≥2 points in PNST).

Loss of appetite (and consequently nutrient deficiency) and unintentional weight loss (leading to a decrease in lean body mass) may be the result of serious disorders such as dysphagia, gastrointestinal symptoms (delayed gastric emptying, constipation, reflux, etc.), depression, or increased caloric demand associated with impaired respiratory efficiency. Therefore, the causes of an unfavorable PNST (or other screening assessment) result should be looked at very carefully.

However, there is no single universally accepted tool for screening nutritional status among pediatric patients and DMD children. The following are used: SGNA—Subjective Global Nutritional Assessment; STAMP—Screening Tool for the Assessment of Malnutrition Pediatrics; PYMS—Pediatric Yorkhill Malnutrition Score; STRONGkids—Screening Tool Risk on Nutritional Status Growth; and PNRS—Pediatric Nutritional Risk Score. Some childhood nutritional screening questionnaires, such as STRONGkids, include questions regarding diseases predisposing children to malnutrition and muscle loss [17,18]. For children with DMD, muscle loss is part of the disease’s natural progression. Such a score categorizes patients as nutritionally at risk, recommending nutritional intervention, weight control twice a week, and weekly nutritional risk assessments, which may be considered excessive for boys with DMD [19]. 

### 4.3. Short Stature

Many studies draw attention to the problem of short stature in boys with DMD. The main cause of stunting growth is chronic steroid therapy [20]. The use of testosterone is becoming more common among boys with DMD. Wood et al. found that this therapy can lead to increased muscle mass and stabilized bone mineral density, reducing the risk of fractures. This resulted in a higher growth rate in boys [21].

### 4.4. Metabolic Parameters

Patients treated with corticosteroids require monitoring of their nutritional status and metabolic parameters. Preventive measures should be introduced to minimize the risk of long-term complications associated with chronic steroid treatment. In this study, a relationship was found between BMI z-score and steroid therapy (boys treated with steroids had a higher BMI z-score), as well as the dose of Delfazacoret and FFMI z-score. Houwen-van Opstal et al. concluded that caloric intake and corticosteroid use were not associated with BMI-z in those in an ambulatory phase; nonetheless, the relationship was reversed for non-ambulatory patients [22]. In our study, ambulatory boys had a lower z-score of BMI than non-ambulatory patients (0.23 vs. 0.92, *p* = 0.04). Cruz-Guzmán et al. showed that excess body mass was associated with being wheelchair-bound regardless of steroid therapy [23]. Similar conclusions were drawn by Bernabe-Garcia et al., who observed that non-ambulant boys consumed 670 kcal more than those of the same age who moved on their own [24]. Thus, controlling caloric intake, especially in boys who have lost mobility, is crucial to reducing the risk of developing obesity. 

Currently, the gold standard for calculating BMR is indirect calorimetry [2]. It needs to be highlighted that total energy expenditure (TEE) also depends on the physical activity level, which equals 1 for non-mobile DMD patients and 1.13 for those with low activity. According to Polish Nutrition Standards [7], the TEE of healthy boys aged 14–17 years with low physical activity is equal to approximately 2600–3000 kcal, while according to our results, the TEE of boys with DMD at the same age with low activity amounts to on average 1697 kcal, and in the case of inactivity, 1502 kcal. Thus, the TEE for most DMD boys is easy to exceed and constitutes a significant risk factor for weight gain [25,26,27]. Due to the fact that the prediction formula takes into account body weight in the calculations, an error (overestimation) may occur. The conclusion is that it is better to use indirect calorimetry, especially in the case of children with DMD and obesity, than prediction formulas. 

On the other hand, the calculated and measured BMR increases physiologically with age, which, in the long run, if the diet is not controlled and the appetite decreases, would cause weight loss and promote cachexia in adults. 

### 4.5. Laboratory Parameters

Malnutrition in DMD is a type of wasting that initially is not associated with severe laboratory disorders. The level of serum albumin and TP remains normal for a long time, and laboratory indicators deteriorate in the state of advanced malnutrition. 

However, we observed vitamin D deficiency (low serum 25-hydroxycholecalciferol 25(OH)D_3_) in as many as 61.4% of patients. Vitamin D is crucial for musculoskeletal and general health. Low levels are associated with an increased risk of death and morbidity, including cardiovascular diseases, infections, and cancer [28]. 

Recommendations suggest the measurement of the level of 25(OH)D_3_ in DMD patients and the administration of cholecalciferol according to the guidelines for the general population. So far, no effective and safe cholecalciferol dosing regimen has been established in the clinical care of DMD patients. From a practical point of view, it is important to promote the use of vitamin D, control its level, and conduct further research on the optimal doses of vitamin D for children with DMD. 

As already mentioned, DMD is associated with the degeneration of muscle mass, and the breakdown process is accompanied by the activation of neutrophils and the production of proinflammatory cytokines. In our study, we did not observe statistically significant differences in the concentration of CRP in individual age groups. There is no consensus on DMD-specific proinflammatory cytokines; undoubtedly, elevated CRP may accompany infection, as in the general population, but it may also be related to excess fat [29]. Perhaps further studies should include the assessment of the concentration of cytokines related to oxidative stress, e.g., myeloperoxidase (MPO) and neutrophil elastase (NE) [30,31]. In comparison with healthy muscle, a biopsy of the dystrophic muscle revealed the overexpression of proinflammatory cytokines such as tumor necrosis factor-alpha (TNF-α), interleukin-1 (IL-1), and interleukin-6 (IL-6) [23,32]. Cruz-Gruzman concluded that DMD subjects with better results on the Vignos Scale had the highest concentrations of IL-1 and TNF-α compared with those with less muscle function. The concentration of proinflammatory cytokines was not associated with nutritional status [33].

### 4.6. Nutritional Management

Bearing in mind that the problem of improper nutritional status is common in the group of patients with Duchenne muscular dystrophy, early implementation of preventive measures in the form of nutritional education, regular monitoring of nutritional status, as well as nutritional treatment in the event of diagnosis of obesity or malnutrition is justified and necessary. There is no single nutritional model recommended for patients with DMD. According to guidelines [2], the goal of nutritional care is to promote a healthy, balanced diet with optimum intake of calories, protein, fluid, and micronutrients, especially calcium and vitamin D. Research conducted on adults with DMD has uncovered an alarming pattern in dietary habits. The findings reveal that a substantial portion of this population fails to adhere to established nutritional guidelines, especially concerning the consumption of essential nutrients. The study particularly emphasizes a widespread shortfall in protein intake along with deficiencies in a range of vitamins and minerals crucial for maintaining health [33].

Nutrition must certainly cover the need for vitamins, minerals, protein, essential fatty acids, fiber, and antioxidants. Petrella C et al. draw attention to the neuroprotective and muscular role of the Mediterranean lifestyle [34]. The Mediterranean diet, rich in phytonutrients, omega-3 fatty acids, monounsaturated fatty acids, magnesium, potassium, calcium, and soluble and water-soluble fiber, reduces the risk of cardiovascular complications and may also reduce the problem of insulin insensitivity [35]. It has a beneficial effect on the intestinal microbiome, contributing to maintaining intestinal integrity [36]. Cedillo et al. proved that the supply of omega-3 fatty acids at a dose of 2.9 g per day reduces the concentration of inflammatory markers [37]. Undoubtedly, other dietary patterns should be considered, such as the anti-inflammatory diet or the DASH diet, a low-glycemic index diet.

The main limitations of our study are the relatively small number of patients (one-center study) and observation at one time point. An additional limitation of this study is the lack of comprehensive tests aimed at identifying deficiencies in individual vitamins and minerals, as well as a detailed examination of patients’ diets in terms of their effectiveness in meeting both caloric and nutritional requirements. 

Further research must focus on assessing the impact of nutritional status on patients’ functional capabilities. This includes accurately determining the impact of malnutrition or specific nutrient deficiencies on physical and cognitive function and examining the benefits that nutritional interventions may provide. This broader focus can provide a more detailed understanding of the direct impact of diet and nutrition on patients’ health and, therefore, offer more effective dietary strategies and interventions to improve overall patient well-being.

On the other hand, the study’s strength was the use of a screening tool for nutritional status assessment together with DXA and anthropometry. Finally, our study provides real-life data reflecting a relatively diverse cohort of DMD patients. 

## 5. Conclusions

Inappropriate nutritional status occurred in almost half of the children with DMD. Excess body weight was the most common abnormality, but also malnutrition was present in 11.8% of patients. The boys with malnutrition or overweight were present in every age group yet with different proportions. It should be noted that the presence of overweight or obesity does not exclude the occurrence of malnutrition. However, the overwhelming majority of irregularities were detected in the 10–13 age group. 

PNST correlated with factors determining nutritional status and could be considered a tool for the screening of malnutrition after testing a larger group of DMD patients.

### Clinical Consequences

The early recognition of nutritional disturbances may be helpful in the prevention of organ and metabolic consequences in DMD patients through the early introduction of proper nutritional support.

## Figures and Tables

**Table 1:** Characteristics of the study group by age categories.

	All (n = 93)	Age Groups (Years)			
		4–6 (n = 38)	7–9 (n = 15)	10–13 (n = 23)	14–17 (n = 17)
**Age (years) mean (range)**	8.54 (4.7–16.9)	5.46 (4.7–6)	8.42 (7.7–8.7)	11.85 (11.5–12.7)	15.0 (14.3–16.9)
Steroid therapy (n)	51	5	15	15	16
Prednisonum (n)	29	5	10	7	7
Deflazacort (n)	22	0	5	8	9
Ambulatory/non-ambulatory (n)	66/27	38/0	13/2	11/12	4/13
Vitamin D supplementation	96%	94.8%	100%	91%	81%
Calcium supplementation	60%	36%	86%	78%	81%

**Table 2:** Results of anthropometry, body composition, and caloric requirements by age groups.

		All (n = 93)	Age Groups (Years)				
			4–6 (n = 38)	7–9 (n = 15)	10–13 (n = 23)	14–17 (n = 17)	*p*-Value
**Anthropometry**							
Weight	kg	34.1 ± 15	19.5 ± 3.4 *	29.7 ± 6.4 ^#^	43 ± 12.5 *	59 ± 15 *^,#^	<0.001
	percentile	40 (17–67)	32 (18–66)	50 (29–78)	49 (17–70)	35 (7–64)	0.464
Height	cm	127 (112–146)	109.7 ± 7 *	127 ± 7.9 ^#^	145 ± 10.9 *^,#^	159.6 ± 8.1 *^,#^	<0.001
	percentile	9 (2.4–34)	13.5(4–41)	15(1–39)	9.7 (2–36)	2 (1–9)	0.098
BMI	kg/m^2^	18.9 ± 4.6	16.10 ± 1.6 *	18.8 ± 3.2	20.8 ± 5.1 *	22.6 ± 5.7 *	<0.001
	percentile	66 (29–67)	56.5 (29–78)	71 (54–94)	81 (18.2–93)	74 (18–96)	0.217
BMI ≥ 85 centile	% of sb	30.0%	13.0%	33.0%	43.0%	35.0%	0.036
BMI < 5 centile	% of sb	11.8%	7.8%	13.3%	17.4%	11.7%	0.483
**Body composition**							
FFM	kg	20.5 (17.1–24.6)	13.9 (12.9–0.9) *	18 (16.2–20.4) ^#^	24.2 (13.5–6.5)	25.6 (24.2–8.3) *^,^*^#^*	0.002
FFMI	kg/m^2^	10.5 (9.8–12.4)	10.9 (10.3–11.4)	10.8 (10.3–12.7)	10.5 (9.5–11.4)	10.2 (9.3–11.7)	0.436
	z-score	−2.4 (−5.5;−1.7)	-	−1 (−1.8;−0.02) *^#^*	−2.43 (−3.2;−1.4)	−5.2 (−6.3;−2.7) *^#^*	0.029
FM	kg	17.3 (9.6–27.5)	5.1 (2.4–6) *	10.1 (7.9–13.2) *^#^*	24.6 (13.5–26.5) *^,#^	34.1 (24.1–41.2) *^,^*^#^*	<0.001
	percentile	87.5 (71–95)	-	85.5 (40–95)	94.0 (75–95.5)	82.1 (70–94.5)	0.833
	z-score	2.11 (1.5–2.4)	-	1.10 (−0.25;1.6) *^#^*	1.54 (0.7–1.7)	1.14 (0.5–1.6) *^#^*	0.009
	%	33.8 ± 8	28.6 ± 2.5 *	35.5 ± 6.8 *^#^*	48.8 ± 9.7^#^	54.2 ± 9.8 *^,^*^#^*	0.002
FMI	kg/m^2^	8.5 (5–14)	3.9 (1.9–4.5)	5.3 (4.7–5.9) *^#^*	11.2 (7.5–13.5)	13.6 (10–16.3) *^#^*	0.002
	z-score	1.58 (0.81–1.9)	-	0.89 (0.28–1.58)	1.57 (0.91–1.87)	1.8 (1.27–1.99)	0.53
Visceral fat mass	g	457.6 ± 252.7	166.7 ± 27.5 *	287.2 ± 76.2 *^#^*	516 ± 169 *	737 ± 319.2 *^,*#*^	<0.001
**Caloric requirements**							
BMR indirect calorimetry	kcal	1353.7 ± 325	1005 ± 194.6 *	1270 ± 254.7	1394 ± 268.7 *	1502 ± 377 *	0.006
BMR kcal/kg of body weight/day	kcal	36.4 ± 12.2	49.5 ± 8.2 *	40.1 ± 6.7	34.1 ± 11.5 *	31.3 ± 13.6 *	0.001
BMR Schofield formula	kcal	1238.5 ± 331	948.5 ± 77.8 *	1178.4 ± 146.5 ^#^	1433.9 ± 221.1 *	1675.1 ± 263.7 *^,*#*^	0.000
**PNST**							
PNST	points	0.55 ± 0.83	0.71 ± 0.83	0.07 ± 0.26	0.56 ± 0.89	0.63 ± 0.95	0.072
PNST ≥ 2 points	% of sb	15.0%	18.0%	0.0%	17.0%	29.0%	0.342

BMI—body mass index; sb—subject; FFM—fat-free mass; FFMI—fat-free mass index; FM—fat mass; FMI—fat mass index; BMR—basal metabolic rate; PNST—Pediatric Nutrition Screening Tool; *^,#^—significant differences between age groups (post hoc analysis).

**Table 3:** Caloric requirements according to calculations and indirect calorimetry depending on BMI percentile classification.

	Underweight (n = 7)	Normal (n = 58)	Overweight (n = 15)	Obesity (n = 13)
BMR based on calorimetry (kcal)	1248.5	1263.5	1303	1553
	(1101–1275.5)	(1029.5–1145.5)	(1167–1580)	(1363–1807)
BMR based on Schofield formula (kcal)	1114,4	1010.2	1273.1	1684
	(868.2–1162.2)	(924.9–1400.9)	(1095–1507)	(1390–2013)
*p*-value	0.067	0.412	0.767	0.028

BMR—basal metabolic rate; results are presented as medians (upper and lower quartiles).

**Table 4:** Comparison of patients by Pediatric Nutrition Screening Tool (PNST).

		PNST < 2 Points (n = 78)	PNST ≥ 2 Points (n = 14)	*p*-Value
Weight	kg	34.6 ± 17.3	28.7 ± 13.9	0.24
	percentile	46.5 (21; 70)	15 (2–37)	0.002
	z-score	−0.08 (−0.78; 0.53)	−1.03 (−2.04; −0.33)	0.002
BMI	kg/m^2^	19.4 ± 4.35	15.2 ± 3.04	<0.001
	percentile	75 (48; 91)	6.5 (1.67; 29)	<0.001
	z-score	0.68 (−0.03; 1.36)	−1.47 (−2.13; −0.53)	<0.001
FFMI	percentile	10441.5 ± 2797	8004 ± 577.9	0.04
	z-score	−2.37 (−3.24; −1.4)	−5.89 (−9.13; −5.54)	0.009
FMI	percentile	10066.8 ± 4776.3	7193.6 ± 5688.5	0.07
	z-score	1.59 (1.07; 1.9)	−0.45 (−0.6; 0.79)	0.003

PNST—Pediatric Nutrition Screening Tool; BMI—body mass index, FFMI—fat-free mass index, FMI—fat mass index.

**Table 5:** The correlations between results of the Pediatric Nutrition Screening Tool (PNST) and selected nutritional status factors.

Nutritional Status Factors & PNST		R	*p*-Value
Age	years	−0.084	0.427
BMI	percentile	−0.544	<0.0001
	z-score	−0.503	<0.0001
FFMI	g/m^2^	−0.502	0.0005
	percentile	−0.375	0.020
	z-score	−0.498	0.001
FMI	g/m^2^	−0.397	0.008
	percentile	−0.346	0.033
	z-score	−0.357	0.027
CRP	mg/L	−0.165	0.341
Albumin	g/L	−0.034	0.748

BMI—body mass index; FFMI—fat-free mass index; FMI—fat mass index; CRP—C-reactive protein.

**Table 6:** Biochemical results by age categories.

	All	Age Groups (Years)				
		4–6 (n = 38)	7–9 (n = 15)	10–13 (n = 23)	14–17 (n = 17)	*p*-Value
Albumin g/L	42.7 ± 6.7	44.2 ± 9.9	43.7 ± 2.7	41.7 ± 2.1	41.8 ± 2.9	0.387
Total protein g/L	71 ± 6.3	71 ± 8	70 ± 5.1	70.2 ± 4.2	72.9 ± 5.1	0.263
Glucose mg/dL	84 ± 8.7	84.8 ± 8.2	84.3 ± 7.3	84.4 ± 11.4	81.5 ± 6.9	0.309
Total cholesterol mg/dL	162.7 ± 30.7	168.4 ± 30.2	158 ± 29.4	166.9 ± 27.8	147.9 ± 33.5	0.134
**LDL cholesterol mg/dL**	97.8 ± 28.9	108.7 ± 30.9	89.5 ± 28	97.9 ± 22	81.5 ± 25.4	0.075
**HDL cholesterol mg/dL**	47.9 ± 10.8	46.4 ± 12.7	47.1 ± 7.5	49.6 ± 8.4	49.7 ± 9.5	0.274
**CRP mg/L**	0.58 (0.4; 1.4)	0.45 (0.4; 0.74) *	0.33 (0.27; 0.4) *^#^*	1.4 (0.9; 5.3)	4.4 (4.1; 8.2) *^,*#*^	0.012
**CPK U/I**	9725.5 (4128; 18,234)	17,500.4 (11,170; 22,769) *	14,884.5 (9724; 18,718) *^#^*	5711 (3019; 8141) *^,*#*^	3226 (2504; 4103) *^,*#*^	<0.001
**LDH U/I**	1012 ± 574.1	1498.1 ± 440.1 *	1177.9 ± 349.7 *^#^*	558.6 ± 170.4 *^,*#*^	403 ± 113.8 *^,*#*^	<0.001
**ALT U/I**	304 (143; 450)	4395 (371; 512) *	358 (275; 547) *^#^*	166 (89; 231) *^,*#*^	68 (54; 126) *^,*#*^	<0.001
**AST U/I**	183 (95; 317)	307 (236; 374) *	261 (182; 356) *^#^*	140 (69; 170) *^,*#*^	75 (57; 93^)^ *^,*#*^	<0.001
**GGTP U/I**	14.6 ± 9	10 ± 2.2 *	12.8 ± 3.3	18.9 ± 12.5	20.6 ± 11.8 *^,*#*^	0.627
**Total bilirubin mg/dL**	0.49 ± 0.3	0.43 ± 0.3 ^*^	0.61 ± 0.3 *^#^*	0.42 ± 0.2	0.6 ± 0.2 ^*^	0.001
**Creatinine mg/dL**	0.21 ± 0.09	0.23 ± 0.1	0.24 ± 0.1	0.19 ± 0.08	0.18 ± 0.06	0.931
**Iron ug/dL**	62 (47.5; 80.5)	61 (44; 81)	60 (49; 98)	61 (45; 80)	63 (60; 67)	0.931
**Ferritin ng/mL**	29.9 (19.1; 45.5)	25.6 (17.8; 33) *	25.4 (18; 36.4)	35.9 (28.8; 47.9)	40.3 (23.6; 76.1) *	0.013
**Calcium mg/dL**	9.8 ± 0.3	9.85 ± 0.3	9.8 ± 0.3	9.6 ± 0.3	9.7 ± 0.4	0.292
**Magnesium mg/dL**	2 ± 0.17	1.9 ± 0.13	2.05 ± 0.3	2.04 ± 0.17	2.0 ± 0.1	0.424
**Vitamin D_3_ 25(OH)D_3_ ng/mL**	23.6 ± 10.5	23.7 ± 9.9	23.3 ± 6.2	23.2 ± 8.4	24.1 ± 11.9	0.879

LDL—low-density lipoprotein, HDL—high-density lipoprotein; CRP—C-reactive protein; CPK—creatine phosphokinase; LDH—lactate dehydrogenase; ALT—alanine aminotransferase; AST—aspartate aminotransferase; GGTP—gamma-glutamyl transferase; *^,#^—significant differences between age groups (post hoc analysis).

## Data Availability

Data are contained within the article.
